# Sexual relationships among men who have sex with men in Hanoi, Vietnam: a qualitative interview study

**DOI:** 10.1186/1471-2458-13-108

**Published:** 2013-02-05

**Authors:** Linus Bengtsson, Anna Thorson, Vu Pham Nguyen Thanh, Peter Allebeck, Rebecca Popenoe

**Affiliations:** 1 Department of Public Health Sciences, Karolinska Institutet, Nobels väg 9, Stockholm, SE-171 77, Sweden; 2 Department of Public Health and Environment, Institute of Sociology, 27 Tran Xuan Soan, Hanoi, Vietnam; 3 Department of Public Health Sciences, Karolinska Institutet, Division of Social Medicine, 17176, Stockholm, Sweden; 4 Division of Nursing, Department of Neurobiology, Caring Sciences and Society, Karolinska Institutet, 23 300, Huddinge, 141 83, Sweden

## Abstract

**Background:**

The prevalence of HIV among men who have sex with men (MSM) in Vietnam’s two largest cities, Hanoi and Ho Chi Minh City, may be above 10%. The aim of this study was to explore sexual relationship patterns and experiences among MSM in Hanoi, to inform HIV preventive efforts. Using purposive sampling we recruited 17 MSM in Hanoi, Vietnam, for in-depth interviews. Participants were aged between 19 and 48 years and came from diverse socio-economic backgrounds. Interviews were tape-recorded, transcribed verbatim, and translated into English. Content analysis was used.

**Results:**

Almost all men in the study saw their same-sex attraction as part of their "nature". Many informants had secret but rich social lives within the MSM social circles in Hanoi. However, poor men had difficulties connecting to these networks. Lifetime sexual partner numbers ranged from one to 200. Seven participants had at some point in their lives been in relationships lasting from one to four years. For several men, relationships were not primarily centered on romantic feelings but instead intimately connected to economic and practical dependence. Sexual relationships varied greatly in terms of emotional attachment, commitment, trust, relationship ideals, sexual satisfaction and exchange of money or gifts. Faithfulness was highly valued but largely seen as unobtainable. Several informants felt strong family pressure to marry a woman and have children.

**Conclusions:**

This study contextualizes sexual relationships among MSM in Hanoi and highlights the extent to which HIV prevention activities need to not only consider HIV prevention in the context of casual sexual encounters but also how to adequately target preventive efforts that can reach MSM in relationships.

## Background

Men who have sex with men (MSM) are at highly increased risk of HIV infection in all global regions [1,2]. Surveys in 2009 among MSM in Hanoi and Ho Chi Minh City in Vietnam have reported HIV prevalence rates of more than ten percent in both cities [3,4]. These high rates of HIV infection have their proximate cause in individual risk behaviors, such as unprotected anal sex and, for a minority of MSM, also injection drug use [5]. These risk factors have been the focus of much research [6-8]. However in order to correctly target preventive measures it is crucial to also acquire a contextualized understanding of the nature of sexual relationships in which these high-risk behaviors occur.

In Vietnam as elsewhere in Asia, MSM identities are based not only on whether a man has sex with men or women, but on a man’s gender role, that is the degree to which he perceives himself as male or female. A man may thus have a masculine role in society and perceive himself as a man but nevertheless have sex with other men, without acquiring a homosexual identity. Other MSM, however, see themselves as fully or partly female and seek male sex partners who are masculine [9,10]. The terms used by MSM themselves thus describe MSM according to the person’s degree of femininity, as well as according to how closeted or open he is. *Bong lo* is the term for men who dress and present themselves as women and are open about their sexual preferences for men. *Bong kin* refers to masculine-looking MSM who are generally not open about their sexual preferences outside the MSM group [10,11]. Identities of MSM in Vietnam are, however, more nuanced and varied than these overarching categories indicate, and Ngo et. al have documented a wide range of terms and categories in use. These include, for example, terms for someone who could be classified as either a man or a woman (*ng*ướ*imang hai dòng máu, h*ờ*t v*ị*tl*ộ*n*) and someone who has sex with both men and women (*hi fi, supersim*). European and American homosexual identities have also impacted on the way young homosexual men construct their identities and the term “gay” has become increasingly common [10].

Though not illegal, homosexuality is heavily stigmatized in Vietnam [11,12]. Until recently homosexuality was not acknowledged publicly at all in Vietnam, but over the last decade effeminate men have become increasingly visible in the streets of Hanoi and Ho Chi Minh City, as have bars and venues specifically catering to MSM [13,14]. Homosexuality had also been given increased attention in newspapers and on TV during the last five years. Although media has often depicted homosexual and transgender men with negative connotations, the attitude lately has been somewhat more positive. Transgender singers such as Thai Tai, Cat Tuyen and Lam Chi Khanh have become popular [15] and recently the first pride parade was held in Hanoi [16].

Research on same-sex relationships that is not solely focused on their link to HIV-related risk behavior has been carried out mostly in high-income countries [17]. These studies show that gay and lesbian couples experience similar levels of relationship satisfaction as heterosexual couples do [17] but some data suggest that relationship stability may be lower than among heterosexual cohabiting couples [18]. We have not found similar research from Vietnam or surrounding countries. Qualitative interview-based studies in China have shown that same-sex relationships are often unstable [19], that concurrent sexual partnerships are common [20], but also that Chinese MSM report substantially fewer sexual partners than MSM in US, Europe and Australia [21].

The aim of this study was to explore sexual relationship patterns and experiences among MSM in Hanoi, a city in major socio-demographic transition like many other cities in East and South-East Asia. Increased knowledge about evolving patterns of relationships among MSM are important to inform prevention efforts and correctly interpret data from quantitative studies.

## Methods

### Sampling and study population

We conducted purposive sampling of 17 men in order to reach MSM from a wide range of backgrounds. Interviews took place between November 2008 and September 2009. Two-thirds of the informants had previously participated in a respondent-driven sampling (RDS) study among MSM in Hanoi [22]. They had, at that time, expressed interest in participating in other research studies and were thus contacted. In order to round out the data with information from MSM who were poorer, older, or had migrant and rural backgrounds, an additional six informants were obtained through snowballing from the first group. Inclusion criteria were person biologically born as males, living in Vietnam, 18 years and above, who had ever had sex (any type) with another man. Eight men from the previous RDS study declined to participate when contacted on the phone. We did not ask for their reason to do so. Participants ranged in age from 19 to 48 years. Half of them were born in Hanoi, and half in villages or smaller towns in northern Vietnam. Six men lived with their parents, one with his sister, one with his wife, two with their boyfriends, three with friends and one man lived alone (Table 1). Three men had children. The men worked as building workers, porters, shop assistants, hair dresser, teacher, medium level managers in private companies, as government employee, computer programmer, two ran their own shops and one was a student.

**Table 1:** Socio-demographic data on the 17 participants

**Variable**	**Category**	**n**
**Age**	18-24	5
	25-34	6
	35-44	2
	45-54	4
	Unassigned	0
**Birth place**	Born in Hanoi	8
	Born outside Hanoi	7
	Unassigned	2
**Living with**	Parent(s)	6
	Sibling(s) only	1
	Friends	3
	Boyfriend	2
	Alone	3
	Unassigned	1
**Education**	12 years or less	7
	More than 12 years	8
	Unassigned	2
**Civil status**	Married (with woman)	2
	Single	14
	Widowed	1
	Unassigned	0
**Income**	2 million VND or less	7
	5 million VND or less	2
	10 million VND or less	2
	10 million VND or more	2
	Unassigned	4

### Interviews

The interviews, which lasted between 40 and 90 minutes, were carried out primarily at the Institute for Studies of Society, Economy and Environment (iSEE), a Hanoi-based Vietnamese research organization working for LGBT rights. When preferred by participants, interviews took place at a café chosen by the informant and in the case of one informant, in his home. 15 interviews were carried out by TPV, a female Vietnamese sociologist, with LB (male Swedish medical doctor) present. LB carried out two interviews with an interpreter, and AT (female Swedish medical doctor) was present at one interview conducted by TPV. All interviews were conducted in Vietnamese. The men were given time to freely express their experiences and thoughts. During most interviews the atmosphere was warm and most men were eager to share their experiences and thoughts. The interviews were tape-recorded, transcribed verbatim, and translated into English. Two translators were used and the translations were checked for accuracy by TPV.

A thematic interview guide was used, focusing on the men’s sexual and love relationships with other men, though the interviews in most cases evolved as informal conversations. In practical terms this meant the informant was asked to relate his experiences of sexual and love relationships from the time when he realized that he was attracted to other men, and up until the present. When the interviewees had been in a large number of relationships, the emphasis was put on those relationships that the participant viewed as being of longer duration or of a deeper emotional significance. Topics emerging from one interview were explored in subsequent interviews.

### Analysis

Content analysis was used. LB and RP performed the initial analysis of the interview transcripts focusing on both preconceived research questions and emergent themes. Research questions included beliefs about, experiences of, and attitudes towards male-male relationships; relationship preferences and perceived advantages and disadvantages to MSM relationship patterns, as well social norms around male-male relationships. An emergent issue during the analyses was the inconsistent statements in many of the interviews. Notes taken during and after the interviews on non-verbal aspects of communication and the “feel” of the interview helped to sort out what these many contradictions meant. During and after the initial analysis of the data the other authors read and commented on the analysis, based on their own readings of the transcripts. Together the authors reached a consensus about how to make sense of and best represent the men’s narratives.

### Ethics

All participants were informed about the content of the study on the phone before coming to the interview location. After reading detailed information about the study, all participants provided written consent or, if they did not want to write their name for reasons of anonymity, they provided clear verbal consent. Participants were not required to reveal any personally identifying information. The study was approved by the Hanoi Medical University Review Board for Bio-Medical Research.

## Results

### Living with one’s “nature”: openness and social context

Almost all men in the study saw their longing to have sex with other men as part of their “nature” (*bảnchất* or *t*ự *nhi*ê*n*) and something they were born with. One man commented, “it is not a disease, it cannot be removed, it is my nature. … For some, this inborn trait was however very difficult to bear. Only one man differed, describing how he had felt forced when he first had sex with another man and worried that this first sexual encounter had somehow changed him into being sexually attracted to men. While the process and experience of coming out varied among the 17 men, only two of the 17 informants had come out completely to their family and only one was completely open to all his social contacts. Most informants thus felt they had to hide important aspects of their life from friends and families, but the majority nonetheless led an active social life within the MSM world, forming and maintaining both sexual relationships and friendships.

Even though two informants described that in Hanoi it was at least possible to “*live with one’s nature*” (*s*ố*ng v*ớ*ib*ả*nch*ấ*tc*ủ*amình*), most informants described themselves as living in two worlds, the non-MSM world and the MSM world, referred to by some as “*our world*” (*gi*ớ*ic*ủ*achúng tôi*), or “*the third world*” (*th*ế *gi*ó*ith*ứ *ba*). As a 23-year old student remarked: “*when I go out with normal friends, I have to be careful when I talk to them … but when I go out with friends in my world, I can talk about what I like, do what I like*”.

This social life, in which many men could be open about their identity, was however much less accessible for three poorer men who had migrated to Hanoi and worked as manual laborers. They had restricted social lives, and because of the demands of their jobs and their small incomes they had fewer opportunities to access places where they could socialize with other MSM. One poorer informant commented:

“As I don't have money, honestly speaking, I am at a great disadvantage. I can't do what I want to. As I don't have friends, my life is miserable…”

Informants described the time up to their middle and late 20s as a period when they could enjoy life after which they experienced heavy social pressure to marry, especially from their parents. Several informants felt a strong pressure to conform to their families’ wishes to continue the male family line by marrying, having children and creating a stable financial base.

### Sexual relationships

Most informants described having had a high number of sexual partners during their lifetimes. Four informants reported ten or fewer sexual partners and seven men described having had more than ten partners. One man described having had approximately 200 life-time partners, while the numbers are unknown for the remaining men. The sexual relationships described varied greatly in terms of emotional attachment, commitment, trust, relationship ideals, sexual satisfaction and exchange of money or gifts. Terms used for relationships and partners were the same as those used in heterosexual relationships, for example boyfriend (*bạn trai*), husband/wife (*chống/vợ*) and lover (người yêu, người tình).

Among the sexually active, four informants had solely casual sexual relationships where emotional attachment to their partners was limited and the sexual encounters took place at a single or at a limited number of occasions with each partner. These informants described the meeting of new partners as a natural urge and as a longing for something new and interesting. After having had sex, this feeling was often followed by boredom and led to the search for new sexual partners. For these informants the sexual partnerships could be both concurrent as well as serial and of short duration.

“I myself am acquainted with many other men. And as I see it, there is the truth that one is dying for something but gets fed up with it very quickly…When I achieve my purpose, I feel bored for some reason. I am 39 years old now and I don't remember how many loves I have had so far in total.”

On the other end of this continuum were four informants who reported that they actively sought stable, monogamous relationships with strong emotional attachment and trust. For them, the sex in itself was less important than the emotional attachment and they were not interested in purely casual sexual encounters. Two of these men were quite well-off, articulate and seemed content with their lives. Two other men were manual laborers from the countryside and were less satisfied with their circumstances. One of these men was depressed and socially isolated while the other man lived in a stable relationship in which his partner had recently become unfaithful.

Half the informants’ relationship lives were located between these extremes of solely practicing casual sex and on the other hand searching only for stable long-term relationships. These men described their relationship experiences as often being of a few months duration interspersed with casual sexual encounters. Frequently these men had occasional, casual sexual encounters also during the time when they were in a stable relationships. Seven of the participants had at some point in their lives been in a relationships lasting from one to four years.

One 22 year-old student captured the experiences of men who often had short but intense and emotionally attached relationships, saying of one love, “We were in love for exactly 18 days.” When asked why the relationship was so short, he answered,

“In our world, there are loves which lasts only 1 or 2 hours…Regarding real love, I haven’t seen any couples in love for over 2 years in Hanoi. Mostly, they are in love for just about 1 or 2 months.”

Many informants who sought, or had previously sought, stable relationships described faithfulness (*lòng chung thủy, sự chung thủy, chung thủy*) as a highly valued quality of a partner, but one which was, however, largely unobtainable. Many men had experienced their partners being unfaithful, many men had themselves been unfaithful and they knew that many of their friends were unfaithful while in relationships. The concept of faithfulness in itself seemed however to be used in the same way as among heterosexuals in Vietnam (i.e. not to have sex with others, and perhaps for some men: not having or acting on certain emotions).

For several of these informants the search and longing for a long-term committed and monogamous relationship had ended in repeated disappointments through a painful process of unfaithfulness and break-ups. This group of men entered the MSM world in search of a romantic, emotionally close and stable relationship but eventually either lost their belief in this goal or chose not to pursue it. As one man commented:

“Regarding all the relationships, I no longer hope for long lasting love, no longer dream of long lasting relationship, I just take it as it comes.

Q: In your opinion, what is an ideal relationship in your world like?

A: The most important thing is faithfulness. But it is very difficult to be faithful in this world, it just exists for a certain period, it cannot exist the lifetime.”

The men described no clear social norms against having too many or too few sexual partners. One man noted that “*only those who are too ugly and unattractive have no choices, and don’t have many lovers*”. Informants did not express any strong norms against unfaithfulness in relationships, accepting it instead as the way things were. “*People in our world are very unfaithful. There are many choices so everyone becomes unfaithful. It is common. One is also a bit angry … but he has to accept*.”

### Sexual preferences

Informants could be broadly divided into those who primarily sought sexual relationships with “*real men*” (*đàn ông đích thực*) and those who sought relationships with men like themselves, i.e. men attracted to other men. The informants defined “*real men*” as men who were heterosexual, not naturally attracted to other men and who were purely masculine. These informants reported finding “*real men”* by approaching financially poor men selling sex or young men in need of material support and a place to live. If these men already had a wife or a girlfriend they were perceived by some informants as more masculine and therefore more attractive. Poor men who could not afford to provide for a real man were seen by these informants as very unfortunate as they instead had to seek out other poor “non-real men” to have sex with.

“You shouldn't think that we are homosexual so we just have sexual relations with homosexual people…I am homosexual but I want to have sex with a real man. If one is homosexual, I won't have sex with him then. I just have friendly relationships, social relationships with gays like me as relationships between people sharing the same plight.”

Other informants, often younger men with university education, distanced themselves from providing for others in exchange for sex, and from wanting to meet a masculine “*real man*”. Instead they sought men, who like themselves, were attracted to other men and several of these men used English terms such as “*gay*”, “*top*” and “*bottom*”.

### Love and money: an inseparable couple

Another more implicit aspect of several men’s understanding of relationships was that love was seldom uncoupled from economic dependence and practical codependence. One informant, the owner of a hair salon, made the point explicitly when answering a question about how he saw his future:

“My future? I just want to live with the one who I consider as a good person…The importance is that I must earn a lot of money… Nobody will sleep with you, marry you, if you don’t have money, even if you are a fashion model.”

This same man went on to say “*regarding love, of course, I treat the one who is kind to me well.*” Another man commented, “*men are just like women – they look for good looks and money in a man.*”

Several men had been left because of another man with a higher status or better financial means. One student commented:

“Maybe that other man is more handsome than me, richer than me, has more expensive motorcycle than mine. Because in this world, love is often measured by motorcycle, cellphone or look. In general, it is appearance. Therefore, I do not believe in anything.”

## Discussion

This study reveals a diverse landscape of sexual relationships among MSM in Hanoi. There was, among the interviewed men, a high prevalence of short relationships, often with concurrent casual partnerships. Unfaithfulness seemed to be frequent and a common reason for break-ups. The men viewed their attraction to other men as part of their nature but for some men this trait was hard to bear. Money, and perhaps more important, the social status that accompanies money, was an important factor shaping sexual and love relationships. The findings may indicate a high frequency of partner change among men in Hanoi. Since condom-use is often more difficult to sustain in emotionally close relationships than in casual sexual encounters [21,23], repeated short-term relationships could be a particularly risky pattern.

Contributing to the pattern of unstable relationships was the pressure almost all men felt from their parents to form a heterosexual family and have children. Some of the men, particularly those who were only sons in their families, felt the desire to carry on the family line. One man also talked about his need to be able to return to his village to be buried when dies. If he were to reveal that he is homosexual, he considered this to not be possible.

Patterns of unstable relationships and difficulties in finding a long-term partner have also been described in studies among urban MSM in Shanghai and Shenzhen, China [19,24]. MSM in Shenzhen described a strong desire to establish a long-term committed and loving relationship but felt that sexual relationships were overemphasized in the MSM community at the expense of more loving ones and that the lack of possibility of same-sex marriage prevented long-term relationships.

The same themes emerged among the men in Hanoi. Some men were actively searching for, or had previously been searching for, stable, monogamous relationships with strong emotional attachment and trust. However, a number of men did not seek exclusive, long-term relationships, but when reflecting on what upholds heterosexual marriages, rather emphasized the role of laws, traditions, economic needs and family pressure. For these men the heterosexual Western ideal of long-term, emotionally committed, exclusive sexual relationships may not be the model against which they measured and judged their own relationships.

There is a close parallel between the men’s views and changing attitudes among young heterosexual men and women in Vietnam. Among young people, perhaps in particular those with good education and income, dating and pre-marital sex have become much more openly discussed and probably more common. This change is also closely connected to a larger emphasis on personal satisfaction than abiding by tradition and the views of the parents [25].

The idea of, and longing for, romantic relationships, which surfaces in many of the interviews, also has it parallel in heterosexual relationships in Vietnam. It developed quickly in Vietnam with the end of the planned economy. Shops catering to young couples planning their weddings have appeared in numerous locations in Hanoi and the young couple in romantic love is probably the most common theme in music, and other media [25,26].

Money also seemed to be an important factor shaping sexual and love relationships. Men with money were perceived as more attractive and several men reported having lost a lover to someone of better means. This pattern may be connected to the increased emphasis on material assets in general in Vietnam, following doi moi, the transformation from a planned to a market economy [25,27]. Another aspect of the importance of money in shaping sexual relationships is sex work. Prostitution and the means to pay for it has increase sharply after the economic reforms [27]. Although male-male sex services are less open and smaller than corresponding female–male sex work, these services are nonetheless highly accessible [28,29] and some men in these interviews regularly and exclusively bought sex from male sex workers.

The study has several limitations. The interview subject was sensitive and social desirability in the discussions may have been present. We perceived however the atmosphere as open and that most participants talked very freely about their thoughts and experiences. Five of the interviewees called, on their own initiative, the main interviewer (TPV) during the weeks after the interviews to continue the conversations. No local MSM were present at the interviews. This may have led to misinterpretation of verbal and non-verbal messages. It may however also have made it possible for men to speak freely about their lives without needing to conform to prevailing norms or fear that their statements would passed on to other MSM. Findings were also discussed with local key informants to avoid misinterpretations of the data*.* The sampling was not probabilistic and the number of informants was limited. Caution should be taken in generalizing the findings to MSM in general. We did not investigate the men’s sexual relationships with women but several nevertheless mentioned sex with women and three had been married. Nonetheless, our study has given insight in a complex area, and we believe the men’s narratives can help increase the understanding of patterns of relationships in this group.

## Conclusion

This study contextualizes sexual relationships among MSM in Hanoi and highlights the extent to which HIV prevention activities need to not only consider HIV prevention in the context of casual sexual encounters but also how to adequately target preventive efforts that can reach MSM in relationships. Legalizing same-sex marriage and a higher cultural acceptance of MSM relationships might help to introduce more stability and permanence into some MSM relationships, thus potentially contributing to decreased HIV transmission.

## Competing interests

The authors declare no competing interests.

## Authors’ contributions

VPT, LB and AT interviewed the participants. LB conceived of the study. LB and RP performed the initial analysis of the interview transcripts. LB drafted the first version of the manuscript. All authors revised the manuscript. All authors read and approved the final manuscript.

## Pre-publication history

The pre-publication history for this paper can be accessed here:

http://www.biomedcentral.com/1471-2458/13/108/prepub

## References

[B1] Baral S Sifakis F Cleghorn F Beyrer C Elevated risk for HIV infection among men who have sex with men in low- and middle-income countries 2000–2006: a systematic review PLoS Med 2007 4 e339 10.1371/journal.pmed.0040339 18052602 PMC2100144

[B2] Beyrer C Baral SD van Griensven F Goodreau SM Chariyalertsak S Wirtz AL Global epidemiology of HIV infection in men who have sex with men Lancet 2012 380 367 377 10.1016/S0140-6736(12)60821-6 22819660 PMC3805037

[B3] Ministry of Health V Integrated Biological and Behavioral Surveillance round 2 Hanoi 2009

[B4] Ministry of Health Vietnam Results from the HIV/STI Integrated Biological and Behavioral Surveillance (IBBS) in Vietnam 2005 – 2006 Hanoi 2006

[B5] Nguyen TA Nguyen HT Le GT Detels R Prevalence and risk factors associated with HIV infection among men having sex with men in Ho Chi Minh City, Vietnam AIDS Behav 2008 12 476 482 10.1007/s10461-007-9267-y 17594139 PMC2903539

[B6] Sun JP Xiao Y Li CM Lu F Allen KL Vermund SH Prevalence and correlates of HIV and syphilis infections among men who have sex with men in seven provinces in China with historically low HIV prevalence J Acquir Immune Defic Syndr 2010 53 S66 S73 20104113 10.1097/QAI.0b013e3181c7db43

[B7] Colby D Minh TT Toan TT Down on the farm: homosexual behaviour, HIV risk and HIV prevalence in rural communities in Khanh Hoa province, Vietnam Sex Transm Infect 2008 84 439 443 10.1136/sti.2008.031294 19028943

[B8] Chemnasiri T Netwong T Visarutratana S Varangrat A Li A Phanuphak P Inconsistent condom use among young men who have sex with men, male sex workers, and transgenders in Thailand AIDS Educ Prev 2010 22 100 109 10.1521/aeap.2010.22.2.100 20387981

[B9] Jenkins C Male Sexuality, Diversity and Culture: Implications for HIV Prevention and Care 2004

[B10] Ngo DA Ross MW Phan H Ratliff EA Trinh T Sherburne L Male homosexual identities, relationships, and practices among young men who have sex with men in Vietnam: implications for Hiv prevention AIDS Educ Prev 2009 21 251 265 10.1521/aeap.2009.21.3.251 19519239 PMC2862473

[B11] Vu BN Girault P Van Do B Colby D Tran LTB Male sexuality in Vietnam: the case of male-to-male sex Sex Health 2008 5 83 88 10.1071/SH07064 18361860

[B12] Vu ML Le Thi MP Nguyen TV Doan KT Tran QT Nguyen TNM MSM in Viet Nam- Social Stigma and Consequences 2009 Hanoi: SHAPC

[B13] Colby D Cao NH Doussantousse S Men who have sex with men and HIV in Vietnam: A review AIDS Educ Prev 2004 16 45 54 10.1521/aeap.16.1.45.27722 15058710

[B14] Berry M Go V Quan V Minh N Ha T Mai N Social environment and HIV risk among MSM in Hanoi and Thai Nguyen 2012 10.1080/09540121.2012.687808 PMC4879820 22624852

[B15] dtinews Transgender in the limelight 2012

[B16] Barton C Gay taboo turns to pride in Vietnam 2012 AFP

[B17] Kurdek LA What do we know about gay and lesbian couples? Curr Dir Psychol Sci 2005 14 251 254 10.1111/j.0963-7214.2005.00375.x

[B18] Andersson G Noack T Seierstad A Weedon-Fekjaer H The demographics of same-sex marriages in Norway and Sweden Demography 2006 43 79 98 10.1353/dem.2006.0001 16579209

[B19] Chapman J Cai YM Hillier S Estcourt C Sex and sexuality in the Shenzhen tongzhi circle: HIV risk context and migrant men who have sex with men in China Cult Health Sex 2009 11 689 702 10.1080/13691050902977562 19551540

[B20] Ha TH Liu HJ Liu H Cai YM Feng TJ Concurrent sexual partnerships among men who have sex with men in Shenzhen, China Sex Transm Dis 2010 37 506 511 20453721 10.1097/OLQ.0b013e3181d707c9

[B21] Guo Y Li XM Stanton B HIV-related behavioral studies of men who have sex with men in China: a systematic review and recommendations for future research AIDS Behav 2011 15 521 534 10.1007/s10461-010-9808-7 21053064 PMC8182773

[B22] Nguyen QC Sexual risk behaviors of men who have sex with men in Viet Nam 2010 Chapel Hill: North Carolina State University

[B23] Chow E Wilson D Zhang L Patterns of condom use among men who have sex with men in China: a systematic review and meta-analysis AIDS Behav 2012 16 653 663 10.1007/s10461-011-9935-9 21461948

[B24] Zhongxin S Farrer J Choi K-h Sexual identity among men who have sex with men in Shanghai China Perpectives 2006 64

[B25] Nguyen P Relationships based on love and relationships based on needs': emerging trends in youth sex culture in contemporary urban Vietnam Mod Asian Stud 2007 41 287 10.1017/S0026749X05002258

[B26] Soucy A Blackburn S Romantic Love andGenderHegemony in Vietnam Love, Sex and Power: Women in Southeast Asia 2001 Clayton: Monash University Press 31 41

[B27] Phinney HM "Rice is essential but tiresome; You should get some noodles": Doi Moi and the political economy of men's extramarital sexual relations and marital HIV risk in Hanoi, Vietnam Am J Public Health 2008 98 650 660 10.2105/AJPH.2007.111534 18309136 PMC2376991

[B28] Clatts M Giang L Goldsamt L Yi H Male sex work and HIV risk among young heroin users in Hanoi, Vietnam Sex Health 2007 4 261 267 10.1071/SH07018 18082070 PMC2718765

[B29] Giang L Clatts M Men selling sex to other men in Hanoi: findings from an ethno-epidemiological study 2009 Hanoi: UNAIDS

